# Relationships between survival and habitat suitability of semi‐aquatic mammals

**DOI:** 10.1002/ece3.6239

**Published:** 2020-04-12

**Authors:** Isidro Barela, Leslie M. Burger, Jimmy Taylor, Kristine O. Evans, Ryo Ogawa, Lance McClintic, Guiming Wang

**Affiliations:** ^1^ Department of Wildlife, Fisheries and Aquaculture Mississippi State University Mississippi State MS USA; ^2^ Siskiyou County Department of Agriculture Yreka CA USA; ^3^ United States Department of Agriculture, Animal Plant Health Inspection Service Oregon Field Station National Wildlife Research Center Corvallis OR USA; ^4^ Present address: United States Department of Agriculture West Virginia Farm Service Agency Romney WV USA

**Keywords:** *Castor canadensis*, fitness, habitat selection, ideal free distribution, maximum entropy, survival‐habitat suitability relationship

## Abstract

Spatial distribution and habitat selection are integral to the study of animal ecology. Habitat selection may optimize the fitness of individuals. Hutchinsonian niche theory posits the fundamental niche of species would support the persistence or growth of populations. Although niche‐based species distribution models (SDMs) and habitat suitability models (HSMs) such as maximum entropy (Maxent) have demonstrated fair to excellent predictive power, few studies have linked the prediction of HSMs to demographic rates. We aimed to test the prediction of Hutchinsonian niche theory that habitat suitability (i.e., likelihood of occurrence) would be positively related to survival of American beaver (*Castor canadensis*), a North American semi‐aquatic, herbivorous, habitat generalist. We also tested the prediction of ideal free distribution that animal fitness, or its surrogate, is independent of habitat suitability at the equilibrium. We estimated beaver monthly survival probability using the Barker model and radio telemetry data collected in northern Alabama, United States from January 2011 to April 2012. A habitat suitability map was generated with Maxent for the entire study site using landscape variables derived from the 2011 National Land Cover Database (30‐m resolution). We found an inverse relationship between habitat suitability index and beaver survival, contradicting the predictions of niche theory and ideal free distribution. Furthermore, four landscape variables selected by American beaver did not predict survival. The beaver population on our study site has been established for 20 or more years and, subsequently, may be approaching or have reached the carrying capacity. Maxent‐predicted increases in habitat use and subsequent intraspecific competition may have reduced beaver survival. Habitat suitability‐fitness relationships may be complex and, in part, contingent upon local animal abundance. Future studies of mechanistic SDMs incorporating local abundance and demographic rates are needed.

## Graphical abstract

We tested the hypothesis that increased habitat suitability would improve American beaver survival. On the contrary, American beaver survival was inversely related to habitat suitability index. Beaver survival was not related to the landscape variables selected by American beaver. Photo credits to “Stockphoto.com”.

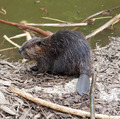

## 1 INTRODUCTION

Habitat suitability models (HSMs) and species distribution models (SDMs) have become popular research tools for spatial ecology, population ecology, and biodiversity conservation (Evcin, Kucuk, & Akturk, 2019; Mohammadi, Ebrahimi, Shahriari Moghadam, & Bosso, 2019; Monsarrat, Novellie, Rushworth, & Kerley, 2019). Although these two models may differ in spatial scopes, with the latter covering a larger spatial extent that may include the entire geographic range of species, HSMs and SDMs are often based on ecological niche theory (Elith & Leathwick, 2009; Hirzel & Le Lay, 2008). The Hutchinsonian ecological niche is the n‐dimensional environmental conditions or hypervolume which supports population persistence (i.e., finite rate of increase *λ* ≥ 1.0) (Hutchinson, 1957) and is most frequently used to conceptualize HSMs or SDMs (Elith & Leathwick, 2009; Hirzel, Hausser, Chessel, & Perrin, 2002; Warren & Seifert, 2011). Under a Hutchinsonian niche approach, measures of individual fitness would be positively related to a habitat suitability index (HSI) score (Holt, 2009; Pironon et al., 2018). However, few studies have investigated relationships between demographic rates (e.g., survival or reproductive rates) and the environmental conditions or landscape variables used to predict habitat suitability (Gaillard et al., 2010; Unglaub, Steinfartz, Drechsler, & Schmidt, 2015).

Under the assumption of ideal free distribution, habitat selection models predict that an animal's spatial distribution is proportional to the amount of resources available in habitat patches and that fitness of individuals is equal among habitat patches at the equilibrium (Fretwell & Lucas, 1969). Empirical data support positive relationships between recruitment or productivity and habitat patch quality in white‐footed mice (*Peromyscus leucopus*) and lions (*Panthera leo*) (Morris & Davidson, 2000; Mosser, Fryxell, Eberly, & Packer, 2009). However, the fitness consequence of habitat suitability may depend on the stage of population dynamics, that is, the initial stage at low abundance versus the equilibrium at carrying capacity (Rosenzweig, 1981). At the initial stage of population growth in relatively low abundance, fitness may be positively related to habitat suitability (Rosenzweig, 1981). As population size approaches carrying capacity, intraspecific competition may be intensified, which would subsequently reduce individual fitness. At equilibrium, habitat patches with higher suitability would support more individuals. Density dependence and dispersal between habitat patches may equalize the fitness of individuals among habitat patches of different quality or suitability as predicted by HSMs (Fretwell & Lucas, 1969; Rosenzweig, 1981).

The American beaver (*Castor canadensis*) is a semi‐aquatic rodent that feeds on deciduous trees, shrubs, and aquatic plants (Baker & Hill, 2003). It is deemed an ecosystem engineer (Jones, Lawton, & Shachak, 1994) because of its substantial impacts on the composition and physiognomy of forest communities and landscapes through herbivory and water impoundment with dam construction (Naiman, Johnston, & Kelley, 1988). Despite these important ecosystem roles, American beaver population dynamics are under‐represented in the literature. We found 14 peer‐reviewed journal articles regarding survival or demography of American beaver in SCOPUS^®^ using the search keywords “American beaver and survival,” “American beaver and demography,” “*Castor canadensis* and survival,” and “*Castor canadensis* and demography” (as of 12 December, 2019). Despite recent studies that investigated habitat selection and habitat suitability of beaver at Redstone Arsenal, a US military installation in northern Alabama, USA (Francis et al., 2017; Wang, McClintic, & Taylor, 2019), relationships between survival and HSI were not examined.

Survival is a critical component and a surrogate of fitness, particularly in organisms with longevity > 1 year (Crone, 2001). Average longevity of American beaver is 10–12 years in the wild (Müller‐Schwarze & Sun, 2003). In this study, we considered survival as a major component of fitness for American beaver. We tested the prediction that beaver survival would be positively related to HSI or beaver‐selected landscape variables (prediction P1). Alternatively, a second prediction (P2) states survival of American beaver would not be related to habitat suitability as predicted by the ideal free distribution model. Since Francis et al. (2017) found that food availability may shape habitat selection by American beaver at both Johnson's (1980) order II (i.e., positioning home ranges across landscapes) and III (i.e., choosing resources within home ranges), we also tested the prediction that survival of American beaver would be positively related to colony‐specific food availability (prediction P3). Although our study focused on American beaver, this study has broad implications for SDMs in general.

## 2 MATERIALS AND METHODS

### 2.1 Study area

**FIGURE 1:**
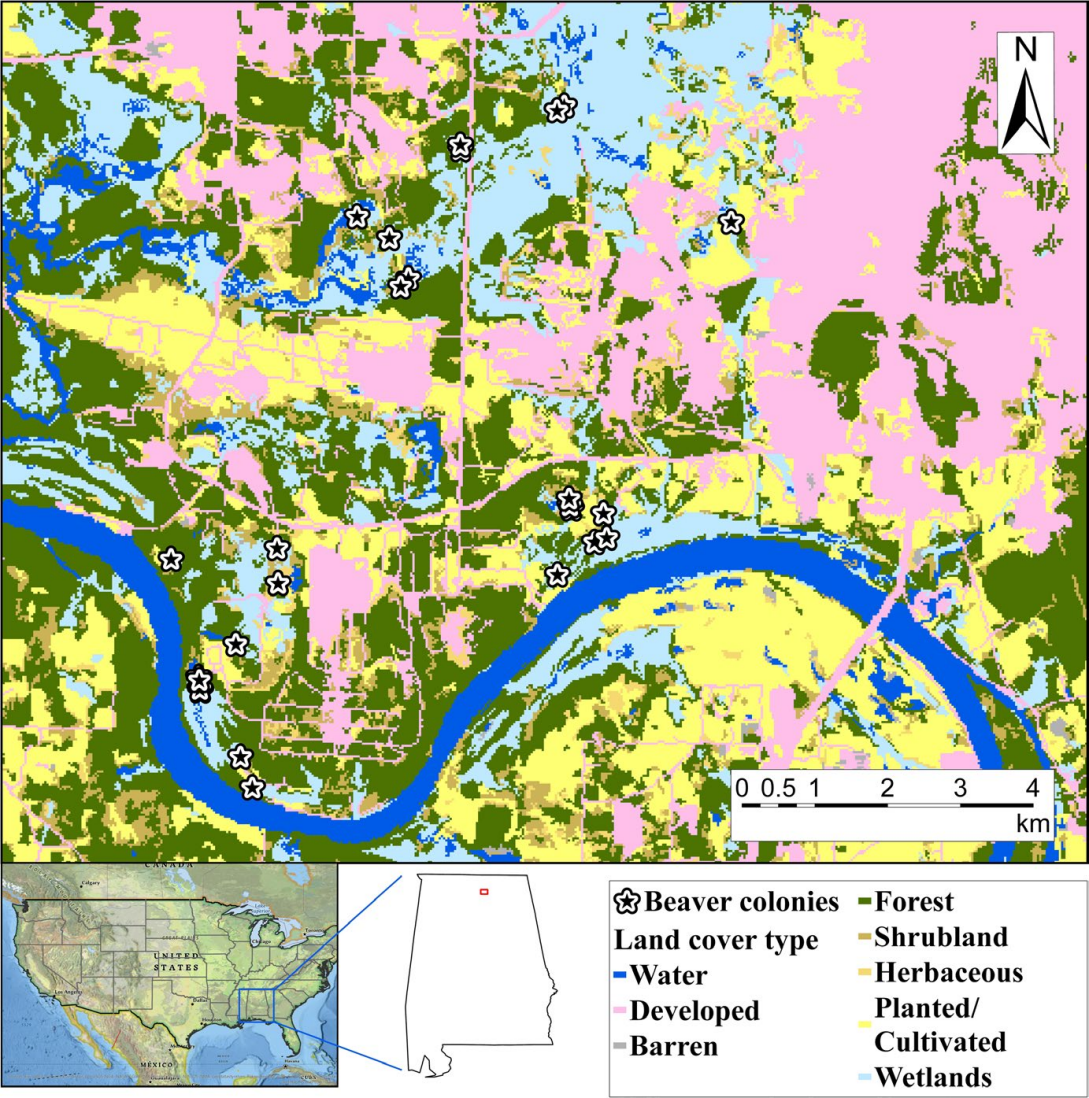
Land use and land cover map of Redstone Arsonal, Alabama, USA. The map was derived from the 2011 National Land Cover Database

We assessed relationships between habitat suitability and beaver survival using radio telemetry data collected from Redstone Arsenal (52°50′–53°86′ E; 38°23′–38°40′ N; hereafter, Redstone; Figure 1) in Madison County, Alabama, USA during 2011–2012 (McClintic, Taylor, Jones, Singleton, & Wang, 2014). Redstone encompasses 15,478 ha of diverse land use and land cover types including agriculture, military test fields, urban centers, bottomland hardwoods, and woody wetlands, upland coniferous forests, mixed forest, and water bodies (wetlands, streams, seasonal swamps, and marshes) (Figure 1; McClintic, Taylor, et al., 2014). Average annual total precipitation ranged from 108 to 180 cm. Monthly temperature averaged 18°C, ranging from 8 to 28°C (Huntsville‐Decatur International Airport weather station, the National Oceanic and Atmospheric Administration ID: 014064).

### 2.2 Beaver capture and telemetry data

We captured American beaver using Hancock live traps (Hancock Trap Company) within Redstone from January to May 2011. We fit a 38‐g (<0.05% of body mass) very high‐frequency (VHF) transmitter (Model 3530, Advanced Telemetry Systems) to each captured subadult (10.9–16.0 kg) and adult (>16 kg) using tail‐mounting methods; juveniles were excluded (Arjo et al., 2008; McClintic, Taylor, et al., 2014). Smith, Windels, Wolf, Klaver, and Belant (2016) demonstrated that tail‐mounting did not affect beaver survival in Minnesota. Capture and handling of beavers was approved by the Institutional Animal Care and Use Committee of the United States Department of Agriculture, National Wildlife Research Center (Protocol No. QA‐1626), and additional details on methodology can be found in McClintic, Taylor, et al. (2014). For survival analysis, we located radio‐tagged beaver once every 4 weeks (i.e., tracking occasions) to determine the fates (i.e., live, dead, undetected, or missing) of radio‐tracked individuals from January 2011 to April 2012. We determined additional information on the fates of tracked beaver from other relocations collected via triangulation between tracking occasions (for home range estimation in a different study) and used those live resighting or dead recovery data for the Barker survival model (Barker, 1997). We located dead beaver as practically possible by triangulation on the VHF mortality signal.

### 2.3 Environmental and landscape variables for estimation of beaver survival

The normalized difference vegetation index (NDVI) is an index of photosynthetic activity and an index of green biomass (Pettorelli, 2013). To assess seasonal variation of vegetation biomass at Redstone, we derived two monthly NDVI time series from 250‐m resolution, 16‐day MODIS (multi‐spectral satellite imagery) using R package *MODIStsp* (Busetto & Ranghetti, 2016). The 250‐m MODIS NDVI is a processed‐ready product. Hourly radio‐tracking demonstrated beaver traveled 0–400 m from their lodge during their daily active hours (McClintic, Wang, Taylor, & Jones, 2014). The 250‐m resolution is about twice the hourly movement distance (112 m/hr) of beaver (Wang et al., 2019); thus, 250‐m spatial resolution is adequate and appropriate for predicting monthly survival. The NDVI time series included: (a) NDVI for Redstone's entire American beaver population for each monthly tracking interval (*popndvi*); and (b) wetland‐ or colony‐specific NDVI for each monthly tracking interval (*colndvi*). We delineated the spatial extent of beaver colonies using a minimum convex polygon from all VHF locations of all radio‐tagged beaver inhabiting a wetland. We averaged NDVI values over all cells or pixels within a colony to estimate colony‐specific NDVI using R packages *raster* and *sp* (Hijmans & van Etten, 2016; Pebesma & Bivand, 2005). If a radio‐tracked individual did not occupy a known colony, we extracted NDVI values by using a circular buffer representing the average spatial extent of beaver colonies. The circular buffer was centered at the centroid of the VHF locations of the individual. Variable *popndvi* was calculated as the average of all *colndvi* values by month. The two NDVI time series were used to predict seasonal survival of beaver.

To evaluate landscape‐beaver survival relationships, we included landscape variables selected by beaver in habitat selection models as predictors of beaver survival. Francis et al. (2017) found American beaver selected woody wetland edge density (m/ha, *wwetbd*), shrub edge density (*shrubbd*), water body edge density (*waterbd*), and relative frequency (0–1.0) of grassland (*grassfq*) out of 30 landscape variables using variable selection with Maxent models and 334 presence locations. Variable selection of Maxent models was carried out with Akaike information criterion (AIC), area under the curve (AUC), and LASSO (Francis et al., 2017). To incorporate landscape features as covariates in the survival models, we derived raster layers for these four landscape variables from the 2011 National Land Cover Database (NLCD) using the program Biomapper (Hirzel et al., 2002). We calculated averages of the four landscape variables for each colony using the same geospatial analysis as we did for NDVI.

To evaluate HSI‐beaver survival relationships, we used the HSI predicted with 15 principal components of 30 landscape variables derived from the 2011 NLCD as a covariate of beaver survival. The HSI map was cross‐validated with a 20:80% testing‐training split of 334 non‐duplicated presence locations (AUC = 0.97) and was further validated using an absence‐free, continuous Boyce index (=0.97) (see Francis et al., 2017 for details). We used the same geospatial analysis for the NDVI to calculate colony‐specific mean HSI from the HSI map. The PCA‐based HSI allowed for minimizing the interdependence between the tests of HSI‐ and landscape‐beaver survival relationships.

### 2.4 Statistical models for monthly survival

American beaver are nocturnal, semi‐aquatic mammals that often swim or live in their dens under water, which affects their detectability by radio telemetry. Consequently, because we were not able to detect all radio‐tagged beaver during each monitoring occasion, we used the Barker model of live captures, live resightings, and dead recoveries within program MARK to estimate monthly survival probabilities (Barker, 1997; White & Burnham, 1999). For the encounter history input, we used monthly live detections (completed during the first week of a monthly interval) of radio‐tagged individuals via VHF telemetry as a live encounter occasion. Live detections occurring anytime between the two successive live encounter occasions within a month were treated as live resightings (Smith et al., 2016). We assumed detection and reporting probabilities were imperfect (<1.0) but constant over time owing to approximately equal radio‐tracking efforts during each live “trapping” occasion. Resighting probability varied with time as a function of VHF relocation efforts (i.e., number of tracking days) during a monthly interval. We assumed constant random migration (immigration = emigration and constant over time) for the parameterization of site fidelity.

We built the Barker models to incorporate colony‐specific landscape variables (*wwetbd*, *shrubbd*, *waterbd*, and *grassfq*) as individual covariates to test predictions P1 and P2 concerning the influences of landscape structure on American beaver survival. Colony‐specific NDVI (*colndvi*) and population NDVI (*popndvi*) were used as an individual covariate and group covariate, respectively, to test prediction P3 regarding the influences of vegetation biomass on beaver survival. We used information‐theoretic approaches to variable selection with AIC corrected for small sample size (AICc) (Burnham & Anderson, 2002). The most approximating model had the lowest AICc values but highest Akaike weight (Burnham & Anderson, 2002). We estimated the variance inflation factor (i.e., median c‐hat) using the most complex model of time‐varying survival (S(time)). If estimated median c‐hat was greater than 1.0, we used quasi‐AICc for small samples (QAICc) and ΔQAICc to select the most parsimonious model and competing models (Burnham & Anderson, 2002; White & Burnham, 1999). The value of ∆QAICc of a model was calculated as the difference in QAICc between the model and the most approximating model (Burnham & Anderson, 2002). A model of ∆QAICc < 2 was considered a competing model of the most approximating model.

Monthly NDVIs represented climate and vegetation seasonality (Pettorelli, 2005). In a preliminary analysis, a model that included seasonal categories (i.e., January–March, April–June, July–September, and October–December) as a covariate did not compete with the model that included *popndvi*. Therefore, to account for seasonal variation in survival, we included monthly NDVIs and colony‐specific landscape variables or colony‐specific HSIs to test predictions P1 and P2.

## 3 RESULTS

**FIGURE 2:**
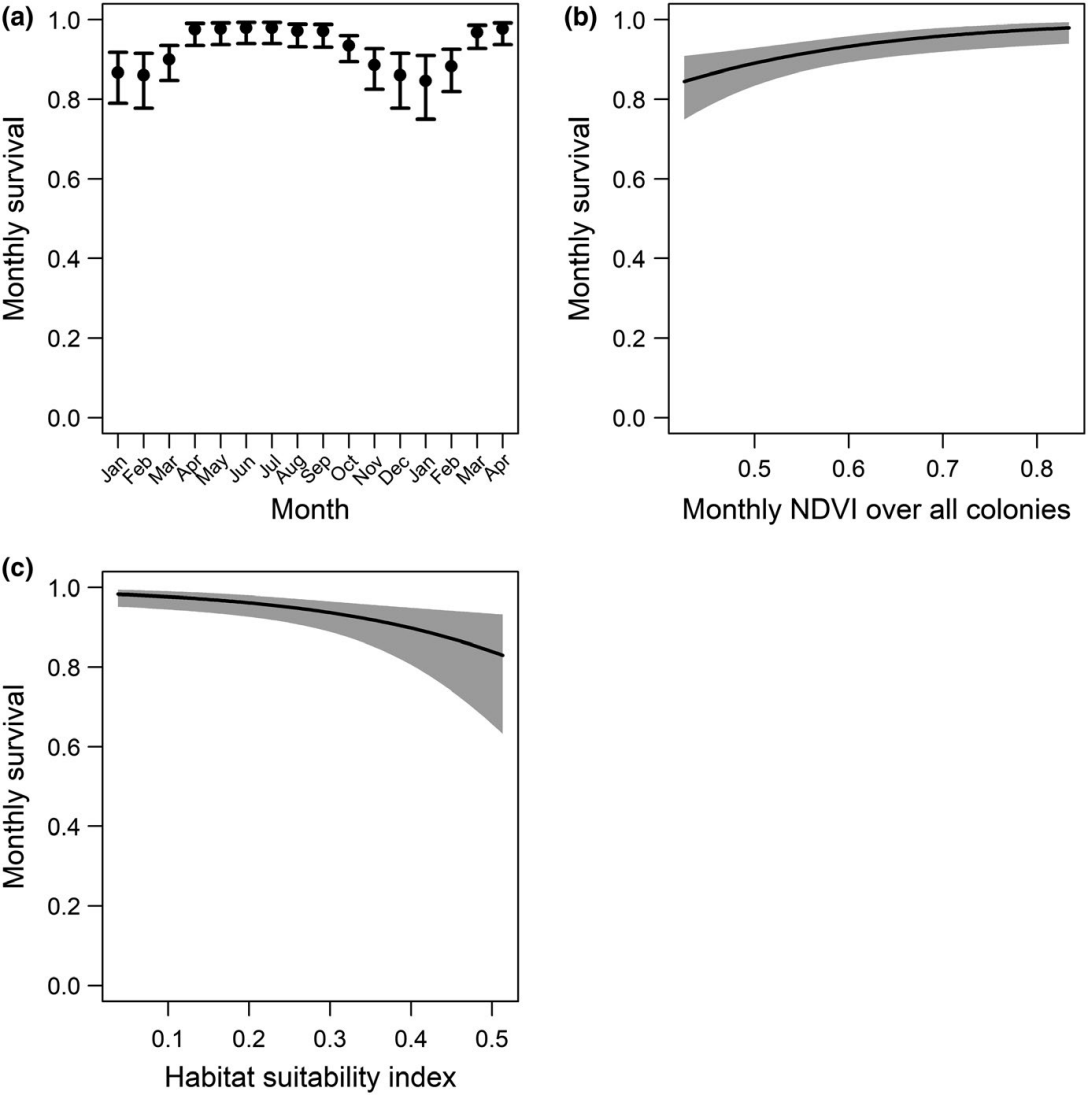
Monthly survival (a) and the effects of normalized difference vegetation index (NDVI) (b) and habitat suitability index (HSI) (c) on survival of American beaver in Redstone Arsenal, northern Alabama, USA from January 2011 to April 2012

We estimated monthly beaver survival for 49 individuals over 16 monthly, live‐trapping occasions. We did not include sex or age as covariates because of our limited sample size and inability to field‐sex captured beaver. Monthly survival estimates from the 49 radio‐tagged beaver were >0.8 over the study period (Figure 2a). Survival exhibited seasonal variation, tending to be lower during December to February than that of the rest of the year (Figure 2a.). Derived annual survival (i.e., product of survival probabilities of 12 consecutive months) ranged from 0.46 to 0.48.

The Barker model using population‐level mean monthly NDVI (*popndvi*) had lower QAICc than those of the Barker model of colony‐level mean monthly NDVI (*colndvi*); therefore, we used *popndvi* as a covariate in subsequent tests of predictions P1–P3. The best approximating survival model including *popndvi* and HSI had an Akaike weight of 0.82, and the ∆QAICc of the second‐best model was 5.99 (Table 1). Therefore, the best model received much more support from the data relative to other 13 candidate models (Table 1). The best models suggested that monthly survival of American beaver was positively related to population‐level NDVI (Table 2, Figure 2b), but was inversely related to HSI, contradictory to prediction P1 (Figure 2c).

**TABLE 1:** Barker's models of monthly survival of American beaver in Redstone Arsenal, Alabama, USA from January 2011 to April 2012

Survival model^a^	QAICc^b^	ΔQAICc	*w*_i_	*K*	QDeviance
popndvi^c^ + hsi^d^	543.74	0.00	0.82	40	458.79
popndvi	549.73	5.99	0.04	39	467.02
popndvi + grassfq^e^	549.97	6.23	0.04	40	465.02
popndvi + wwetbd^f^	550.50	6.76	0.03	40	465.55
colndvi^g^	550.73	6.99	0.03	39	468.02
popndvi + waterbd^h^	550.96	7.22	0.02	40	466.01
popndvi + shrubbd^i^	551.97	8.22	0.01	40	467.01
hsi	553.17	9.43	0.01	39	470.46
null	559.05	15.31	0.00	38	478.59
grassfq	559.81	16.07	0.00	39	477.11
wwetbd	560.02	16.28	0.00	39	477.32
waterbd	560.52	16.78	0.00	39	477.81
shrubbd	561.26	17.52	0.00	39	478.55
time	574.51	30.77	0.00	53	459.69

Note

^a^ Survival model indicates the covariate(s) of survival probability in the Barker model.

^b^ QAICc is quasi Akaike information criterion, *w*_i_ the Akaike weight of model i, ΔQAICc is the difference in QAICc between a model and the lowest QAICc value, and QDeviance is quasi deviance of survival models. Letter *K* is the number of known parameters.

^c^ Covariate symbol popndvi stands for population‐level monthly mean normalized difference vegetation index (NDVI).

^d^ HSI average habitat suitability index.

^e^ Grassfq colony‐specific relative frequency of grassland.

^f^ wwetbd colony‐specific mean edge density of woody wetland.

^g^ Colndvi colony‐specific monthly mean NDVI.

^h^ waterbd colony‐specific mean edge density of water bodies.

^i^ Shrubbd colony‐specific mean edge density of shrub.

Word “time” stands for time‐varying survival and “null” for constant survival over time.

**TABLE 2:** Coefficient estimates of the most approximating Barker model of monthly survival of American beaver in Redstone Arsenal, Alabama, USA from January 2011 to April 2012

Variable	Estimate	*SE*	LCI	UCI
intercept	0.734	1.089	−1.401	2.869
popndvi	5.371	1.773	1.896	8.847
hsi	−5.249	1.852	−8.880	−1.619

Note

*SE* is standard error, LCL the lower limit of 95% confidence interval (CI), and UCL the upper limit of 95% CI. popndvi stands for population‐level mean normalized difference vegetation index (NDVI) and hsi average habitat suitability index.

## 4 DISCUSSION

Animals select habitat to optimize their resource use with fitness consequences (Fretwell & Lucas, 1969; Rosenzweig, 1981). It has long been recognized that neither presence nor abundance is an appropriate indicator of habitat quality (Gaillard et al., 2010; Johnson, 2007; Van Horne, 1983). Our study is among few studies that have tried to link habitat suitability indices to demographic rates (Gallien, Münkemüller, Albert, Boulangeat, & Thuiller, 2010; Monnet, Hardouin, Robert, Hingrat, & Jiguet, 2015; Unglaub et al., 2015). Our results did not support the prediction (P1) that survival of American beaver would be positively related to HSI. Additionally, the inverse relationship between survival and habitat suitability did not support the prediction (P2) that survival of American beaver was not related to HSI. However, the findings of this study supported the prediction (P3) that survival of American beaver would be positively related to NDVI. Our findings suggest that numeric predictors of correlative HSMs may not predict survival and fitness consequences of space use of semi‐aquatic mammals.

As an obligate herbivore, American beaver select high‐quality habitat to maximize energy intakes (Gallant et al., 2016). Increases in green plant biomass may enhance beaver survival during spring and summer, whereas lack of green plant biomass during winter, along with cold temperatures, may reduce their survival. Seasonal variation in beaver survival is supported by our monthly survival estimation at Redstone Arsenal and by previous research in southern Illinois, United States (Bloomquist & Nielsen, 2010). Population‐level monthly mean NDVI was the only time‐varying covariate in the best model; therefore, the positive effects of monthly NDVI reasonably represented seasonal variation in survival, suggesting that seasonal variation in food availability may result in seasonal variation in beaver survival.

American beaver survival on Redstone Arsenal was consistent with observed geographic variation in other US populations, albeit at the relatively low end of the reported range. Derived annual survival of American beaver was about 0.47 on our study site, similar to estimates observed in east‐central Illinois (0.28–0.59) (Havens, Crawford, & Nelson, 2013) and Wyoming (0.43) (McKinstry & Anderson, 2002) but lower than estimates observed in southern Illinois (0.76 for females and 0.87 for males) (Bloomquist & Nielsen, 2010), Massachusetts (0.84) (DeStefano, Koenen, Henner, & Strules, 2006), and Minnesota (0.77) (Smith et al., 2016). Geographic differences in survival may be caused by differences in land cover, land use, and hydrologic connectivity among different sites in addition to variation in beaver control measures (e.g., dam removal) that impact survival and space use in the environment. It is uncertain whether variation in climate affects survival as demonstrated by widely varying estimates of annual survival in east‐central versus southern Illinois. However, it was shown that variation in precipitation and temperature impacted young of the year, juvenile, and dominant adult survival in Eurasian beaver (*Castor fiber*) in Norway (Campbell, Nouvellet, Newman, Macdonald, & Rosell, 2012).

Despite a positive finite rate of increase suggested by Hutchinsonian ecological niche theory, relationships between HSI, abundance, and demographic rates appear to be complex (Bacon et al., 2017; Dallas & Hastings, 2018). Unglaub et al. (2015) found that HSI was positively related to reproduction but not survival of the Great Crested Newt (*Triturus cristatus*). Postrelease survival of captive‐bred North African Houbara Bustards (*Chlamydotis undulata undulata*) was greater in habitat with a high HSI than that within habitat with a low HSI (Monnet et al., 2015). Postrelease growth of translocated populations in previously “vacant” habitat may not have reached equilibrium, and thus, survival may be positively related to HSI. In contrast to the Bustards, American beaver populations in Redstone have been established for 20 or more years. It is plausible that this population has reached carrying capacity, and survival in more suitable habitat is being reduced by intraspecific competition or density dependence at higher abundance. Interestingly, the relationship between HSI and North African Houbara Bustard daily nest survival from February to June changed progressively from an inverse to a positive relation over 12 years (Bacon et al., 2017). Inconsistent links between habitat suitability and demography among studies and temporal variation in the relationships warrant future studies to investigate relationships among habitat suitability, demography, and abundance.

Both proximate and ultimate factors influence behavioral decisions of animals (Krebs & Davies, 1984). Animals may use environmental conditions or variables such as landscape structure as habitat cues which may have fitness consequences to animals (Gilroy & Sutherland, 2007). For example, daily nest survival of white‐headed woodpeckers (*Picoides albolarvatus*) was related to nest HSI and density of large trees, a key variable of nest habitat selection by the woodpecker (Hollenbeck, Saab, & Frenzel, 2011). Our data did not support links between survival and the landscape variables selected by American beaver. Selection of those landscape variables or structure may not result in an increase in beaver survival. This finding suggests the uncertainty of fitness or demographic consequences of habitat selection estimated by Maxent models.

Correlative species distribution or resource selection models have the advantage of convenience in location data collection (e.g., with the aid of GPS tracking or biologging technologies), remote sensing of environmental conditions, and various powerful statistical toolboxes and packages for model development (Jarnevich, Stohlgren, Kumar, Morisette, & Holcombe, 2015). However, correlative models may not distinguish between cause and effect of resource use (Gaillard et al., 2010; Meineri, Deville, Grémillet, Gauthier‐Clerc, & Béchet, 2015). For instance, American beaver fell trees and cut seedlings to build dams, which impound water and create ephemeral, herbaceous wetlands (Collen & Gibson, 2000). Water impoundment, bark stripping, and logging by beaver may create forest openings and increase the amounts of forest, shrub, and water body edges (Townsend & Butler, 1996). The lack of association between beaver survival and edge density may indicate that the positive correlation between fine‐scale habitat use and edge densities is a by‐product of engineering activities rather than habitat selection. Future studies that use long‐term, time series analysis of fine‐resolution, remote sensing data to detect beaver colonization, dam construction, and landscape impacts will help clarify the influence of landscape characteristics on beaver population demographics (Martin, Jasinski, Kendall, Dahl, & Hyndman, 2015; Tape, Jones, Arp, Nitze, & Grosse, 2018).

We demonstrated an inverse relationship between survival of American beaver and HSI. Additionally, our data did not support links between beaver survival and four landscape variables selected by beaver. Our findings reiterate the long‐recognized need for process‐based models such as spatially‐explicit, individual‐based models, and capture‐recapture models that unify landscape ecology, demography, and habitat selection (Meineri et al., 2015; Royle, Fuller, & Sutherland, 2018). This new line of research will require collection of long‐term, spatial data on animal demography, environments, and movements.

## CONFLICT OF INTEREST

The authors have no conflict of interest related to this work.

## AUTHOR CONTRIBUTIONS

**Isidro Barela:** Data curation (equal); formal analysis (equal); investigation (equal); writing – original draft (supporting); writing – review and editing (equal). **Leslie M. Burger:** Conceptualization (equal); funding acquisition (equal); investigation (equal); supervision (lead); writing – original draft (supporting); writing – review and editing (equal). **Jimmy Taylor:** Conceptualization (supporting); data curation (equal); funding acquisition (supporting); investigation (equal); resources (equal); supervision (supporting); writing – review and editing (equal). **Kristine O. Evans:** Investigation (equal); resources (equal); supervision (supporting); writing – original draft (supporting); writing – review and editing (equal). **Ryo Ogawa:** Data curation (equal); formal analysis (equal); software (equal); writing – review and editing (equal). **Lance McClintic:** Data curation (equal); investigation (equal); writing – review and editing (equal). **Guiming Wang:** Conceptualization (equal); formal analysis (equal); funding acquisition (supporting); investigation (equal); methodology (lead); resources (supporting); supervision (supporting); validation (lead); writing – original draft (lead); writing – review and editing (equal).

## Data Availability

Radio telemetry data and data on habitat suitability index and landscape variable used for survival models used in this study are available in Dryad (https://doi.org/10.5061/dryad.t4b8gthzd).
